# Wastewater-Based Surveillance of Antimicrobial Resistance in Niger: An Exploratory Study

**DOI:** 10.4269/ajtmh.23-0204

**Published:** 2023-08-28

**Authors:** Sani Ousmane, Issifi A. Kollo, Ronan Jambou, Rakia Boubacar, Ahmed M. Arzika, Ramatou Maliki, Abdou Amza, Zijun Liu, Elodie Lebas, Emily Colby, Lina Zhong, Cindi Chen, Armin Hinterwirth, Thuy Doan, Thomas M. Lietman, Kieran S. O’Brien

**Affiliations:** ^1^ Centre de Recherche Médicale et Sanitaire, Niamey, Niger;; ^2^ Centre de Recherche et Interventions en Santé Publique, Birni N’Gaoure, Niger;; ^3^ Programme Nationale de Santé Oculaire, Niamey, Niger;; ^4^ Francis I. Proctor Foundation, University of California, San Francisco, California;; ^5^ Department of Ophthalmology, University of California, San Francisco, California;; ^6^ Department of Epidemiology and Biostatistics, University of California, San Francisco, California;; ^7^ Institute for Global Health Sciences, University of California, San Francisco, California

## Abstract

Wastewater-based surveillance is increasingly recognized as an important approach to monitoring population-level antimicrobial resistance (AMR). In this exploratory study, we examined the use of metagenomics to evaluate AMR using untreated wastewater samples routinely collected by the Niger national polio surveillance program. Forty-eight stored samples from two seasons each year over 4 years (2016–2019) in three regions were selected for inclusion in this study and processed using unbiased DNA deep sequencing. Normalized number of reads of genetic determinants for different antibiotic classes were compared over time, by season, and by location. Correlations in resistance were examined among classes. Changes in reads per million per year were demonstrated for several classes, including decreases over time in resistance determinants for phenicols (–3.3, 95% CI: –8.7 to –0.1, *P* = 0.029) and increases over time for aminocoumarins (3.8, 95% CI: 0.0 to 11.4, *P* = 0.043), fluoroquinolones (6.8, 95% CI: 0.0 to 20.5, *P* = 0.048), and beta-lactams (0.85, 95% CI: 0.1 to 1.7, *P* = 0.006). Sulfonamide resistance was higher in the post–rainy season compared with the dry season (5.2-fold change, 95% CI: 3.4 to 7.9, *P* < 0.001). No differences were detected when comparing other classes by season or by site for any antibiotic class. Positive correlations were identified in genetic determinants of resistance among several antibiotic classes. These results demonstrate the potential utility of leveraging existing wastewater sample collection in this setting for AMR surveillance.

## INTRODUCTION

Antimicrobial resistance (AMR) surveillance is essential to monitor trends and target interventions. Estimates suggest that the burden of morbidity and mortality associated with AMR is disproportionately borne by low- and middle-income countries (LMICs), with west Africa estimated to face the highest mortality associated with AMR.^1^ At the same time, AMR data tends to be particularly sparse from LMICs,^1^^,^^2^ underscoring a need to support enhanced surveillance capacity in these settings. Networks like the Global Antimicrobial Resistance Surveillance System (GLASS) aim to address the need for increased global surveillance, although much of the current focus is on individual-level surveillance in healthcare settings,^3^^,^^4^ which may bias estimates and fail to capture fully the population-based nature of AMR prevalence and spread.^5^^–^^7^ In addition, phenotypic methods are typically used in these settings,^3^^,^^4^^,^^6^ restricting surveillance to prespecified, culturable species.^8^

Metagenomic deep sequencing of wastewater has been proposed as a complement to traditional surveillance approaches.^9^ Wastewater is a known reservoir for AMR and a potential incubator for selection of antimicrobial resistance and horizontal gene transfer.^7^^,^^10^ Wastewater-based surveillance has the potential to be an efficient, passive, and population-based monitoring system, enabling non-intrusive capture of community-level AMR.^9^ Evidence suggests that wastewater surveillance of AMR is highly concordant with human surveillance for both phenotypic and genotypic approaches.^11^^,^^12^ The use of metagenomics for wastewater surveillance is appealing because it circumvents the limitations of the targeting required by both phenotypic approaches and polymerase chain reaction (PCR).^10^ Increasingly, calls are being made for increased use of high-throughput sequencing with urban wastewater for monitoring in LMICs,^9^^,^^13^ with an emphasis on the acceptability and economic feasibility of these methods.

In this exploratory study, we examined the use of high-throughput sequencing to monitor AMR from wastewater in Niger by leveraging sample collection routinely conducted by the national polio surveillance program. We aimed to determine the following: 1) whether unbiased DNA deep sequencing (DNA-seq) could be used with existing untreated wastewater collection to monitor genetic determinants of AMR over time, 2) whether this approach could detect differences in AMR gene abundance by location or season, and 3) the presence of correlation in genetic determinants of AMR across antibiotic classes.

## MATERIALS AND METHODS

### Study design and setting.

This retrospective exploratory study used wastewater samples collected throughout Niger as part of a polio surveillance program. Samples were processed with DNA-seq to examine relationships in genetic determinants of antibiotic resistance by time, location, and season and to explore the correlation across different classes of antibiotics.

In November 2015, the World Health Organization (WHO) introduced environmental surveillance as part of the polio eradication program in Niger. Eight sentinel sites from three distinct geographic parts of the country were selected for inclusion in the program, including four sites in the capital of Niamey, two sites in the Maradi region, and two sites in the Diffa region. The sites are located in densely population urban areas with more than 100,000 inhabitants that have sewage systems with a permanent flow to enable active poliovirus detection. As of April 2016, these sites were fully functional, collecting and sending two sewage samples twice each month to the national public health laboratory, the Center de Recherche Médicale et Sanitaire (CERMES). Two additional sentinel sites from the region of Zinder were added in 2019.

### Sample collection and management.

At each site, wastewater collections were conducted between 6:00 and 6:30 am to ensure the wastewater streams were relatively free from solid debris and detergents. The sample collectors wore personal protective equipment, including laboratory coats, tall boots, hand gloves, and face masks. Grab sampling methods were used for collections. The sample collectors used a clean bucket attached to a rope and immersed it into the middle of flowing untreated wastewater. Once filled, the bucket was brought back up and a funnel was used to transfer 2 L of wastewater from the bucket into a clean polystyrene container with a double closure. The sample collector disinfected the container and placed the clean container into a cooler equipped with frozen ice packs. The cooler was then sealed with adhesive tape and immediately sent via common transport bus to CERMES, where the specimens were concentrated according to WHO guidelines.^14^

After transportation to CERMES, 500 mL of wastewater was centrifuged, at 1,500 × *g* for 20 minutes. The pellet was stored at 4°C for later use. The supernatant pH was adjusted to neutral (pH 7–7.5) using NaOH or HCl, accordingly. Adjusted supernatant was mixed with 35 mL of sodium chloride 5 M, 287 mL of polyethylene glycol 6000 at 29% (i.e., 60 g/L) and 39.5 mL of Dextran T40 at 22%. The mixture was kept in constant agitation for 1 hour at 4°C using a magnetic stir plate then poured into the separating funnels, which were left to stand overnight at 4°C to allow phase separation. The concentrate was harvested by collecting the lower layer and the interphase slowly drop-wise, and this concentrate was mixed with the pellet. Then chloroform was added up to 20% of the total volume of concentrate plus pellet. A few grams of glass beads were added to the mixture, then it was shaken vigorously before centrifuging at 1,500 × *g* for 20 min. Final concentrate volume collected was between 10 and 15 mL of the upper phase. Concentrate samples were aliquoted in 5-mL cryotubes and stored at –80°C. Approximately 10 mL of the final concentrate was sent to the WHO regional polio laboratory for poliovirus detection process,^14^ and at least 4 mL of same concentrate was stored locally.

For the present study, a total of 48 samples from those stored locally were selected to represent a range of the available years, seasons, and locations. From each of the three regions with collection since 2016 (Niamey, Maradi, Diffa), samples from two sites were selected from two seasons in each of the 4 years available at the time of the study (2016–2019). Maradi and Diffa collections each took place at two collection sites, and Niamey collections took place at four sites, so the two Niamey sites with the greatest sample availability were selected for inclusion in this project. Seasons were defined based on sample availability across time periods roughly corresponding to the dry season (March–April, labeled “Spring” in results) and post–rainy season (October–December, labeled “Fall” in results). The post–rainy season was chosen based on the availability of samples in these months and the mass distribution of sulfonamides as part of seasonal malaria chemoprevention during the rainy season. Samples were shipped to the University of California, San Francisco at 2–8°C in October 2020 and stored at –80°C until processing.

### Sample processing and bioinformatics.

All samples were deidentified. Laboratory personnel were masked to sample year and location. Samples were processed in a random manner. DNA was extracted using the ZymoBioMICS DNA/RNA Miniprep kit per manufacturer's recommendations (Zymo Research, Irvine, CA). Ten microliters of extracted DNA were prepared for sequencing on the Illumina sequencing platform using the NEBNext Ultra II DNA Library Prep Kit (New England Biolabs, Ipswich, MA) and then amplified with 21 PCR cycles. Libraries were then pooled and sequenced with the use of Illumina NovaSeq 6000 (Illumina, San Diego, CA) with 150-nucleotide paired-end sequencing.

Sequencing reads were processed as previously described.^15^ Briefly, host-reads were removed with the remaining non–host reads filtered for quality. Those reads passing quality filter were then aligned to the MEGARes reference antimicrobial database, version 1.0.1, using Burrows–Wheeler Aligner with default settings. Matched antimicrobial genetic resistance determinants grouped at the class-level and subjected to further statistical analyses.

### Statistical methods.

Analyses were conducted using normalized number of reads of genetic determinants for different antibiotic classes. Linear regression was used to examine normalized read number by continuous time, with a separate model used for each class and permutation used to estimate *P* values. DESeq2 was used to compare class-level normalized read number by season with a Benjamini–Hochberg adjustment using a false discovery rate of 5%.^16^^,^^17^ To compare number of reads by site, we used analysis of variance for each antibiotic class, with pairwise comparisons conducted for any class with an omnibus test *P* value of < 0.05. Spearman correlation coefficients were estimated for the correlation of genetic determinants of resistance for each pair of antibiotic classes. Overall resistome structure and diversity were examined by season and site using Manhattan distance with PERMANOVA and Shannon diversity index with Kruskal–Wallis tests, respectively. Given the exploratory nature of the research questions, adjustments for multiple comparisons were not performed.

## RESULTS

**Figure 1.**
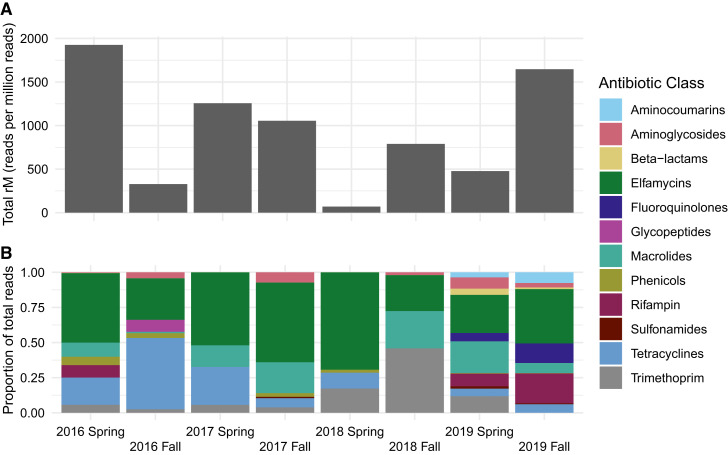
Summary of yield by season and year from unbiased DNA deep sequencing of wastewater samples from Niger, 2016–2019. (**A**) The total number of reads of genetic determinants of resistance per million reads for all classes for each season and year. (**B**) The relative abundance (proportion of total reads) of genetic determinants of resistance for each antibiotic class.

Figure 1 summarizes the yield from samples pooled by season and year. Overall, reads of genetic determinants of resistance to different antibiotic classes varied across each set of samples, such that not all classes were detected in each sample by site or season (Figure 1, Supplemental Table 1, Supplemental Figures 1–3). Genetic resistance determinants were most commonly detected in eflamycin, tetracycline, macrolide, and rifampin antibiotic classes. Samples from 2019 produced resistance determinants to a greater number of antibiotic classes compared with earlier years.

**Figure 2.**
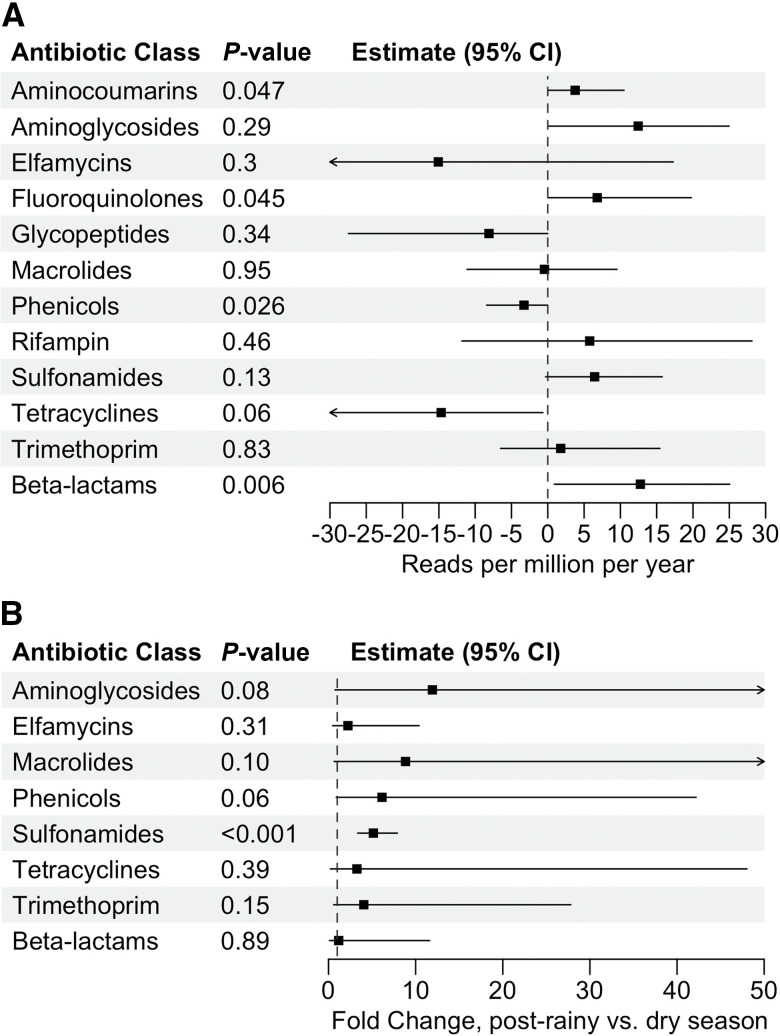
Forest plots of change in reads of genetic determinants of resistance for each antibiotic class over time (**A**) and season (**B**) in wastewater samples from Niger, 2016–2019. (**A**) Absolute change in normalized reads per million per year as estimated with linear regression. (**B**) Relative change in normalized reads per million by season as estimated using DESeq2. Arrows indicate confidence bounds that extend beyond the axis presented.

Figure 2A and B display normalized read number of genetic determinants of resistance to different antibiotic classes by calendar time and by season, respectively. Figure 2A suggests decreasing resistance to phenicols over time (–3.3 reads per million per year, 95% CI: –8.7 to –0.1, *P* = 0.029) and increasing resistance to aminocoumarins (3.8 reads per million per year, 95% CI: 0.0–11.4, *P* = 0.043), fluoroquinolones (6.8 reads per million per year, 95% CI: 0.0–20.5, *P* = 0.048), and beta-lactams (0.85 reads per million per year, 95% CI: 0.1–1.7, *P* = 0.006). Sulfonamide resistance was higher in the post–rainy season compared with the dry season (Figure 2B; 5.2-fold change, 95% CI: 3.4–7.9, *P* < 0.001). No other differences were detected when comparing by season. No differences were detected by site for any antibiotic class (Supplemental Table 1). No differences were detected in overall resistome structure or diversity by season or site (Supplemental Figures 1 and 2).

**Figure 3.**
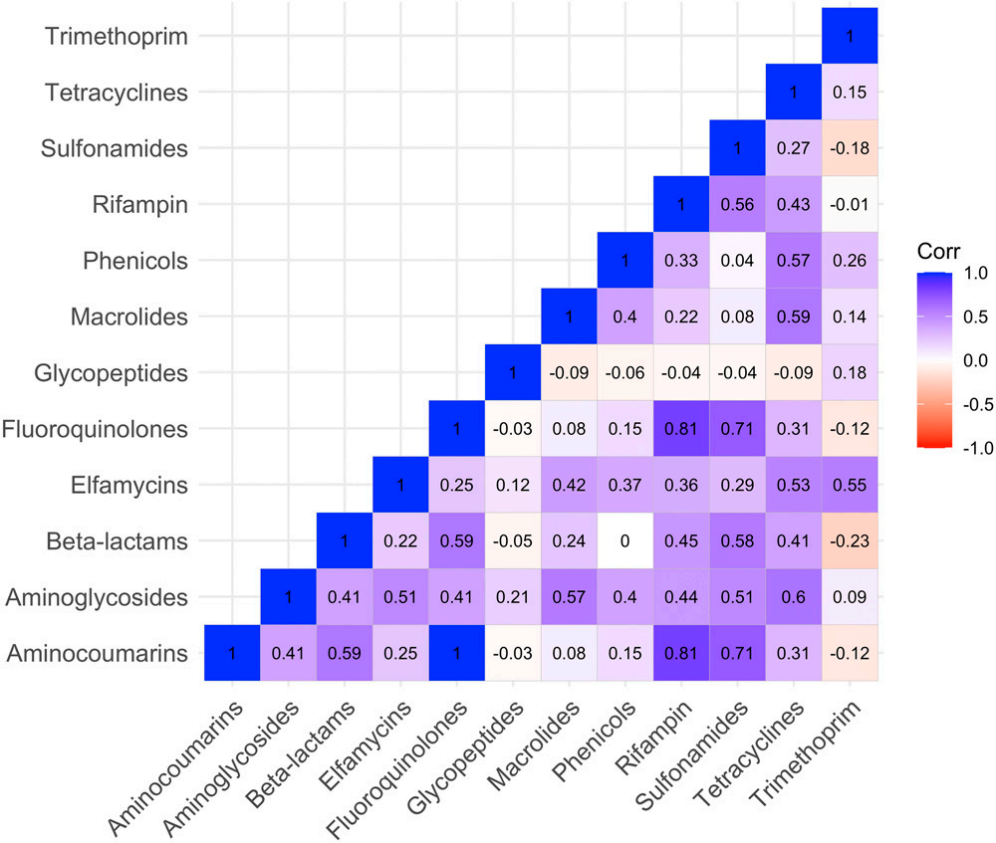
Correlation between normalized number of reads of genetic determinants of resistance to different antibiotic classes from wastewater samples in Niger, 2016–2019. Correlations estimated using Spearman’s correlation coefficient. Corr = correlation.

Figure 3 shows the correlation among genetic determinants of resistance to different antibiotic classes. Of note, correlations > 0.7 were seen between both rifampin and sulfonamides with aminocoumarins and fluoroquinolones. Correlations > 0.55 were also demonstrated between beta-lactams and aminocoumarins, fluoroquinolones, and sulfonamides, as well as between tetracylines and aminoglycosides, macrolides, and phenicols.

## DISCUSSION

Wastewater is increasingly recognized as an important reservoir of resistance genes that enables conditions conducive to selection pressure and resistance gene transfer. Here, we aimed to explore the use of high-throughput sequencing to monitor AMR in wastewater across multiple sites in Niger, leveraging existing wastewater collection conducted by the national polio program. Overall, we found relative stability in the resistome by time, season, and location, consistent with similar genomic analyses of global sewage-based surveillance of AMR.^18^ We did see changes in genetic determinants of resistance to a few specific antimicrobial classes over time that align with known shifts in antibiotic consumption. Others have also found that patterns were dependent on specific classes but not seen with overall resistome structure or diversity.^18^ We found an overall lack of seasonal relationships, similar to a prior study that found limited impact of seasonality on resistome diversity.^18^ A notable exception was sulfonamide resistance, for which our results suggested a seasonal pattern consistent with mass distribution of seasonal malaria chemoprevention (SMC) during the late summer/early fall rainy season. Prior studies have also documented positive correlations in resistance to different antibiotic classes, which may be related to correlated patterns of increasing antibiotic use across classes as well as several potential epidemiological and genetic causes, including biochemical mechanisms that confer resistance to multiple classes, genetic linkage of resistance determinants, and bystander selection, among others.^19^^–^^23^

Increased antibiotic consumption has been associated with increased AMR in numerous settings.^24^^–^^26^ Evidence suggests increasing antibiotic consumption in LMICs.^27^^,^^28^ In sub-Saharan Africa, studies suggest the continued and even increased use of penicillins and fluoroquinolones since 2000,^28^ consistent with the increases we saw here in beta-lactam and fluoroquinolone resistance over time. Studies have also noted a greater burden of sulfonamide resistance in African countries relative to those in Europe and North America.^28^ Although we were unable to detect an increase over time, our results suggested a seasonal increase in sulfonamide resistance, which may correspond to the seasonal distribution of sulfonamides as part of SMC.^29^ SMC expanded in Niger during the study period, becoming widespread in Niger by 2019.^29^^,^^30^ The decreases in phenicol resistance seen in our study might be associated with global reductions in its routine use associated with the risk of aplastic anemia.^31^

Strengths of this study include the use of DNA-seq, an agnostic approach that allows for the exploration of thousands of antibiotic resistance genes simultaneously. In addition, wastewater is representative of the general population, enabling a community-level understanding of AMR without the biases inherent in healthcare-based sampling approaches that focus on patients presenting with infection. Limitations of this study include the small number of samples overall and the lack of data on location- and time-specific antibiotic use patterns for comparison. We did not detect resistance determinants to all classes of interest from all samples by site or time/season and we are not able to determine whether this is due to the small sample size alone or true variation, though this will be important for future research to inform surveillance approaches. Yield tends to be low with low biomass samples like the ones used in this study, and those reported here are in line with those reported elsewhere for similar samples.^18^ In addition, generalizability of these results will be limited to urban areas in West Africa with wastewater available. In settings like this in Niger, many rural areas will not have wastewater available for sampling.

Overall, this exploratory study demonstrated the potential utility of leveraging existing wastewater collection in West African setting to comprehensively monitor AMR.

## Supplemental Materials


Supplemental materials

## References

[1] Murray CJL , 2022. Global burden of bacterial antimicrobial resistance in 2019: a systematic analysis. Lancet 399: 629–655. 35065702 10.1016/S0140-6736(21)02724-0 PMC8841637

[2] Tadesse BT Ashley EA Ongarello S Havumaki J Wijegoonewardena M González IJ Dittrich S, 2017. Antimicrobial resistance in Africa: a systematic review. BMC Infect Dis 17: 616. 28893183 10.1186/s12879-017-2713-1 PMC5594539

[3] World Health Organization , 2020. Global Antimicrobial Resistance Surveillance System (GLASS) Report: Early Implementation 2020. Geneva, Switzerland: WHO.

[4] World Health Organization , 2022. Global Antimicrobial Resistance Surveillance System (GLASS) Report 2022. Geneva, Switzerland: WHO.

[5] Olesen SW Lipsitch M Grad YH, 2020. The role of “spillover” in antibiotic resistance. Proc Natl Acad Sci USA 117: 29063–29068. 33139558 10.1073/pnas.2013694117 PMC7682407

[6] World Health Organization , 2018. Global Antimicrobial Resistance Surveillance System (GLASS) Report: Early Implementation 2017–2018. Geneva, Switzerland: WHO.

[7] Fahrenfeld N Bisceglia KJ, 2016. Emerging investigators series: sewer surveillance for monitoring antibiotic use and prevalence of antibiotic resistance: urban sewer epidemiology. Environ Sci Water Res Technol 2: 788–799.

[8] Tacconelli E Sifakis F Harbarth S Schrijver R van Mourik M Voss A Sharland M Rajendran NB Rodríguez-Baño J; EPI-Net COMBACTE-MAGNET Group , 2018. Surveillance for control of antimicrobial resistance. Lancet Infect Dis 18: e99–e106. 29102325 10.1016/S1473-3099(17)30485-1

[9] Hendriksen RS , 2019. Global monitoring of antimicrobial resistance based on metagenomics analyses of urban sewage. Nat Commun 10: 1124. 30850636 10.1038/s41467-019-08853-3 PMC6408512

[10] Karkman A Do TT Walsh F Virta MPJ, 2018. Antibiotic-resistance genes in waste water. Trends Microbiol 26: 220–228. 29033338 10.1016/j.tim.2017.09.005

[11] Chau KK , 2022. Systematic review of wastewater surveillance of antimicrobial resistance in human populations. Environ Int 162: 107171. 35290866 10.1016/j.envint.2022.107171 PMC8960996

[12] Karkman A Berglund F Flach C-F Kristiansson E Larsson DGJ, 2020. Predicting clinical resistance prevalence using sewage metagenomic data. Commun Biol 3: 711. 33244050 10.1038/s42003-020-01439-6 PMC7692497

[13] Bastaraud A Cecchi P Handschumacher P Altmann M Jambou R, 2020. Urbanization and waterborne pathogen emergence in low-income countries: where and how to conduct surveys? Int J Environ Res Public Health 17: 480. 31940838 10.3390/ijerph17020480 PMC7013806

[14] World Health Organization , 2003. Guidelines for Environmental Surveillance of Poliovirus Circulation. Geneva, Switzerland: WHO Department of Vaccines and Biologicals.

[15] Doan T , 2020. Macrolide and nonmacrolide resistance with mass azithromycin distribution. N Engl J Med 383: 1941–1950. 33176084 10.1056/NEJMoa2002606 PMC7492079

[16] Love MI Huber W Anders S, 2014. Moderated estimation of fold change and dispersion for RNA-seq data with DESeq2. Genome Biol 15: 550. 25516281 10.1186/s13059-014-0550-8 PMC4302049

[17] Benjamini Y Hochberg Y, 1995. Controlling the false discovery rate: a practical and powerful approach to multiple testing. J R Stat Soc B 57: 289–300.

[18] Munk P , 2022. Genomic analysis of sewage from 101 countries reveals global landscape of antimicrobial resistance. Nat Commun 13: 7251. 36456547 10.1038/s41467-022-34312-7 PMC9715550

[19] Chang HH Cohen T Grad YH Hanage WP O’Brien TF Lipsitch M, 2015. Origin and proliferation of multiple-drug resistance in bacterial pathogens. Microbiol Mol Biol Rev 79: 101–116. 25652543 10.1128/MMBR.00039-14 PMC4402963

[20] Lipsitch M, 2001. Measuring and interpreting associations between antibiotic use and penicillin resistance in *Streptococcus pneumoniae.* Clin Infect Dis 32: 1044–1054. 11264033 10.1086/319604

[21] Pouwels KB Muller-Pebody B Smieszek T Hopkins S Robotham JV, 2019. Selection and co-selection of antibiotic resistances among *Escherichia coli* by antibiotic use in primary care: an ecological analysis. PLoS One 14: e0218134. 31181106 10.1371/journal.pone.0218134 PMC6557515

[22] Pouwels KB Freeman R Muller-Pebody B Rooney G Henderson KL Robotham JV Smieszek T, 2018. Association between use of different antibiotics and trimethoprim resistance: going beyond the obvious crude association. J Antimicrob Chemother 73: 1700–1707. 29394363 10.1093/jac/dky031

[23] Donskey CJ Chowdhry TK Hecker MT Hoyen CK Hanrahan JA Hujer AM Hutton-Thomas RA Whalen CC Bonomo RA Rice LB, 2000. Effect of antibiotic therapy on the density of vancomycin-resistant enterococci in the stool of colonized patients. N Engl J Med 343: 1925–1932. 11136263 10.1056/NEJM200012283432604 PMC4370337

[24] Bell BG Schellevis F Stobberingh E Goossens H Pringle M, 2014. A systematic review and meta-analysis of the effects of antibiotic consumption on antibiotic resistance. BMC Infect Dis 14: 13. 24405683 10.1186/1471-2334-14-13 PMC3897982

[25] Olesen SW Barnett ML MacFadden DR Brownstein JS Hernández-Díaz S Lipsitch M Grad YH, 2018. The distribution of antibiotic use and its association with antibiotic resistance. eLife 7: e39435. 30560781 10.7554/eLife.39435 PMC6307856

[26] Goossens H Ferech M Vander Stichele R Elseviers M, 2005. Outpatient antibiotic use in Europe and association with resistance: a cross-national database study. Lancet 365: 579–587. 15708101 10.1016/S0140-6736(05)17907-0

[27] Klein EY Van Boeckel TP Martinez EM Pant S Gandra S Levin SA Goossens H Laxminarayan R, 2018. Global increase and geographic convergence in antibiotic consumption between 2000 and 2015. Proc Natl Acad Sci USA 115: E3463–E3470. 29581252 10.1073/pnas.1717295115 PMC5899442

[28] Browne AJ , 2021. Global antibiotic consumption and usage in humans, 2000–18: a spatial modelling study. Lancet Planet Health 5: e893–e904. 34774223 10.1016/S2542-5196(21)00280-1 PMC8654683

[29] ACCESS-SMC Partnership , 2020. Effectiveness of seasonal malaria chemoprevention at scale in west and central Africa: an observational study. Lancet 396: 1829–1840. 33278936 10.1016/S0140-6736(20)32227-3 PMC7718580

[30] Koko DC Maazou A Jackou H Eddis C, 2022. Analysis of attitudes and practices influencing adherence to seasonal malaria chemoprevention in children under 5 years of age in the Dosso Region of Niger. Malar J 21: 375. 36474264 10.1186/s12936-022-04407-z PMC9727940

[31] Schilling CG Larson TA Uden DL, 1988. Chloramphenicol-associated aplastic anemia. J Pharm Technol 4: 54–59.

