# Supplementary material for: Wastewater-Based Surveillance of Antimicrobial Resistance in Niger: An Exploratory Study
Source: Am J Trop Med Hyg. 2023 Aug 28;109(4):725–9. doi: 10.4269/ajtmh.23-0204 (PMC10551091; doi:10.4269/ajtmh.23-0204)

SUPPLEMENTARY MATERIAL

Supplementary Table1. Summary of results of comparison of mean reads per million by site.[1]

Antibiotic class	Diffa Koura 1 (Diffa)	Diffa Koura 3 (Diffa)	Gao Barki (Maradi)	Soura Bildi (Maradi)	Musee National du Niger (Niamey)	Stade Seyni Kountche (Niamey)	*P*-values
Aminocoumarins	0.00	0.00	0.00	2.18	0.00	15.69	0.46
Aminoglycosides	4.72	1.02	0.66	14.91	2.14	2.23	0.13
Beta-lactams	0.51	0.31	0.54	1.58	1.37	1.00	0.93
Elfamycins	60.67	2.57	43.23	97.97	98.05	112.65	0.39
Fluoroquinolones	0.00	0.00	0.00	3.52	0.00	28.62	0.46
Glycopeptides	3.51	0.00	0.00	0.00	0.00	0.00	0.43
Macrolides	40.07	0.00	20.41	62.33	9.28	0.00	0.08
Phenicols	0.19	0.00	11.60	7.66	0.86	0.00	0.42
Rifampin	0.00	0.00	0.00	26.69	0.00	43.82	0.48
Sulfonamides	1.40	0.00	0.00	0.87	0.00	1.36	0.64
Tetracyclines	43.23	0.75	21.71	61.92	6.11	1.40	0.30
Trimethoprim	2.51	43.80	1.83	1.85	23.92	8.38	0.54

[1]Table columns are labeled with site name and region in parentheses.
[2]*P*-values estimated using ANOVA.

Supplementary Figure 1. Overall resistome structure summarized as Manhattan distance and compared by site (A) and season (B) using PERMANOVA.

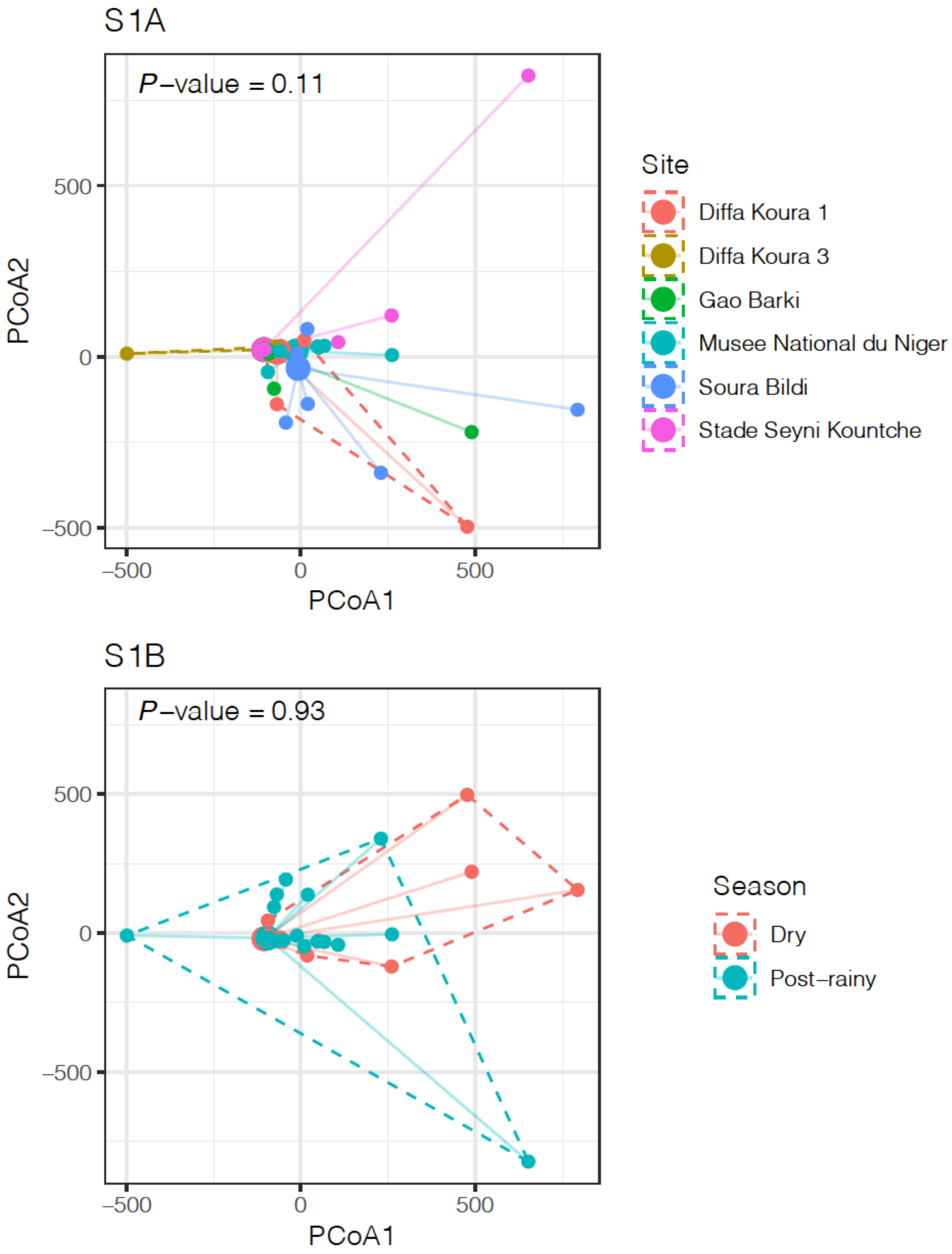

Supplementary Figure 2. Overall resistome diversity summarized using Shannon diversity index and compared by site (A) and season (B) using Kruskal Wallis tests.

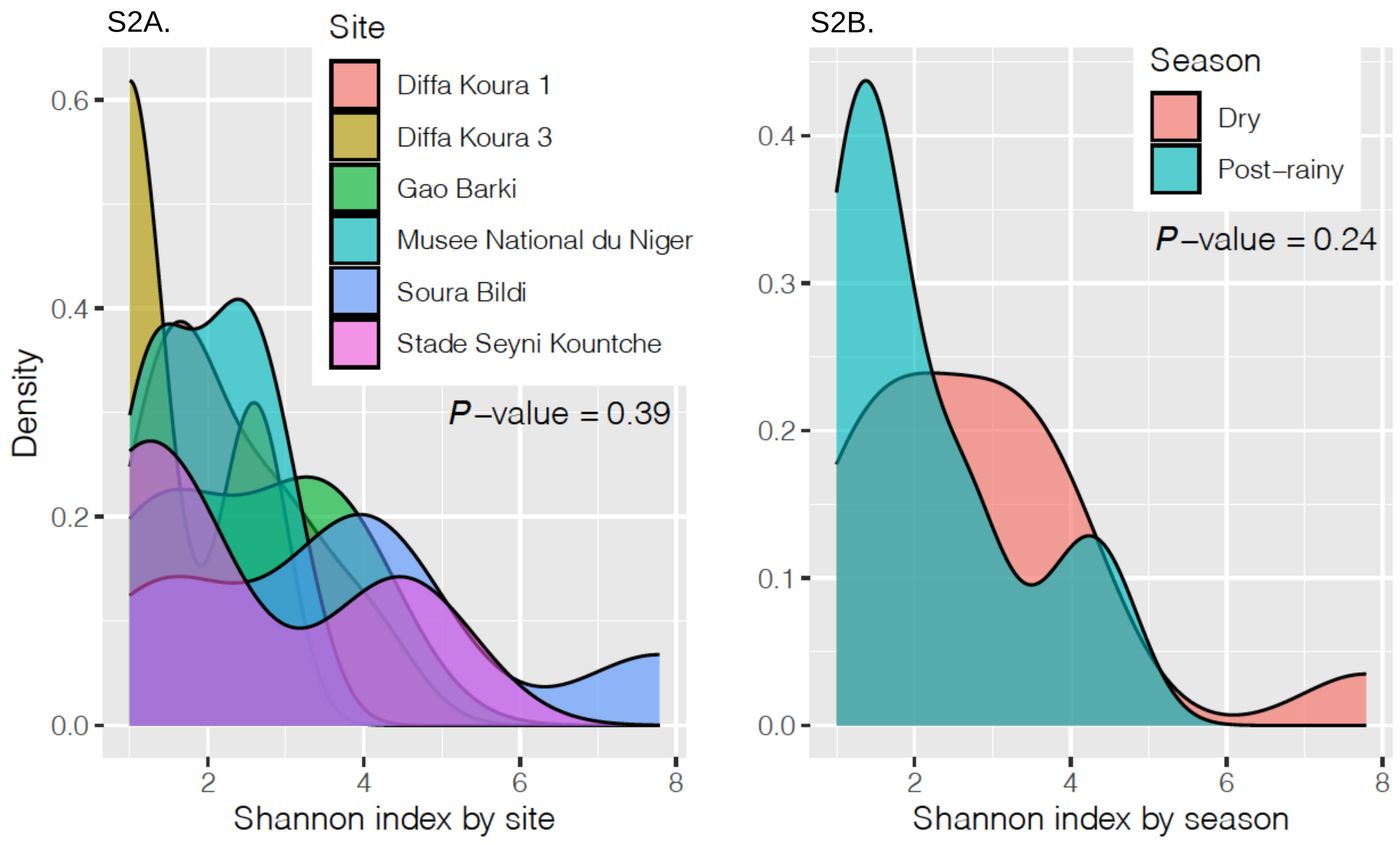

Supplementary Figure 3. Summary of yield by season and year for each site from unbiased DNA deep sequencing of wastewater samples from Niger, 2016-2019. In each panel, the top figure shows the total number of reads of genetic determinants of resistance per million reads for all classes for each season and year. The bottom figure displays the relative abundance (proportion of total reads) of genetic determinants of resistance for each antibiotic class. Figures S3A-S3F display results for each site, Diffa Koura 1 (Diffa), Diffa Koura 3 (Diffa), Gao Barki (Maradi), Soura Bildi (Maradi), Musée National du Niger (Niamey), and Stade Seyni Kountche (Niamey), respectively.

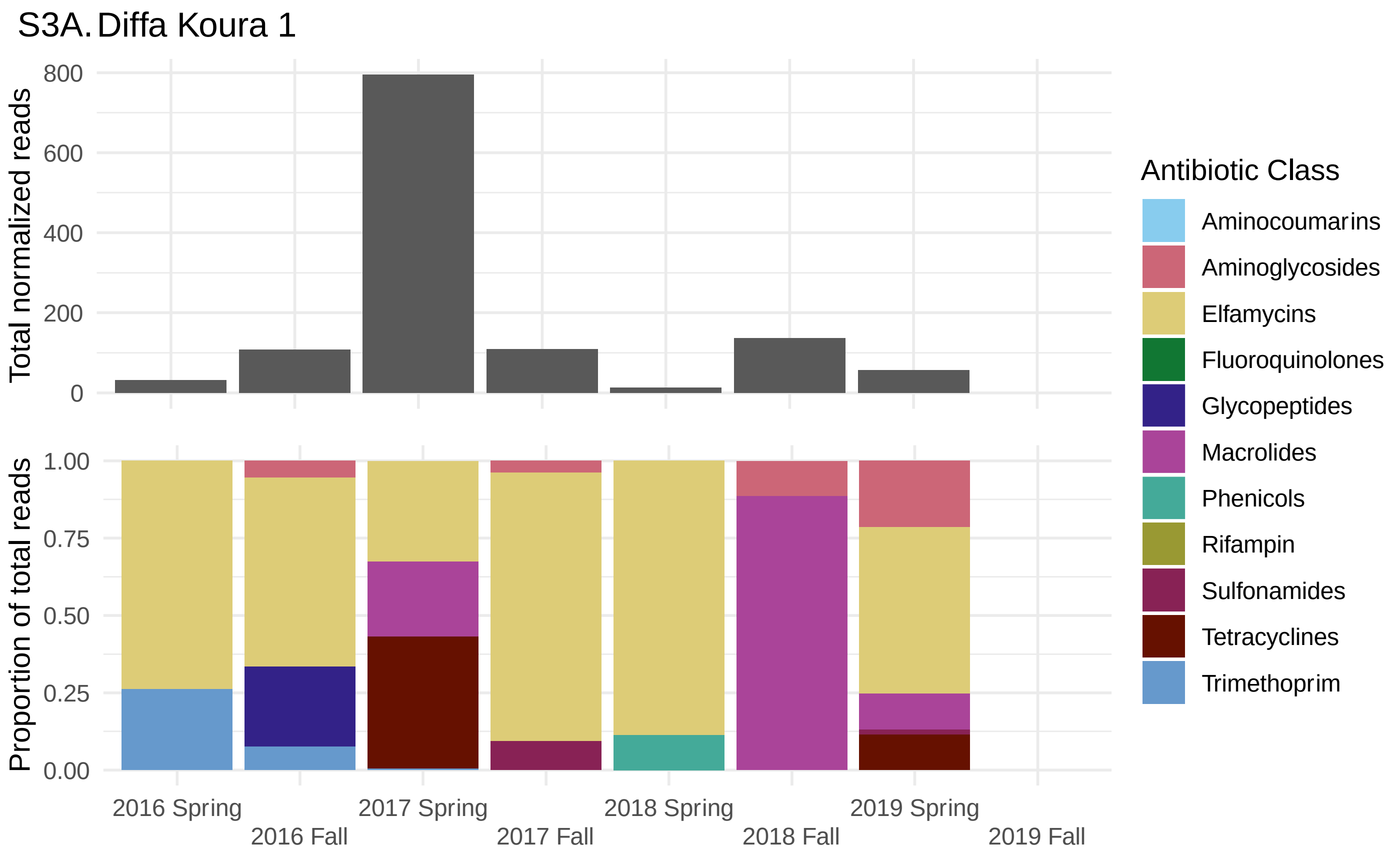

S3B. Diffa Koura 3

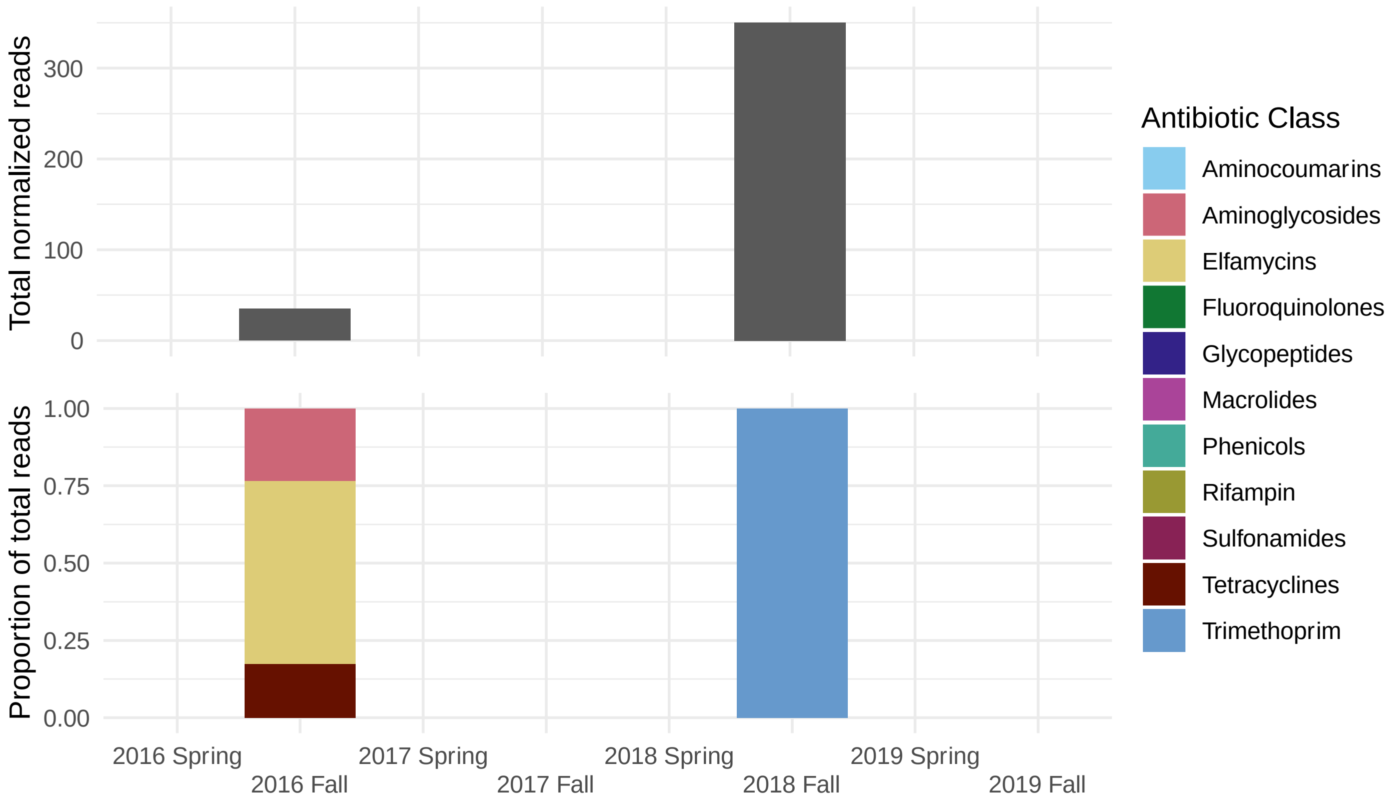

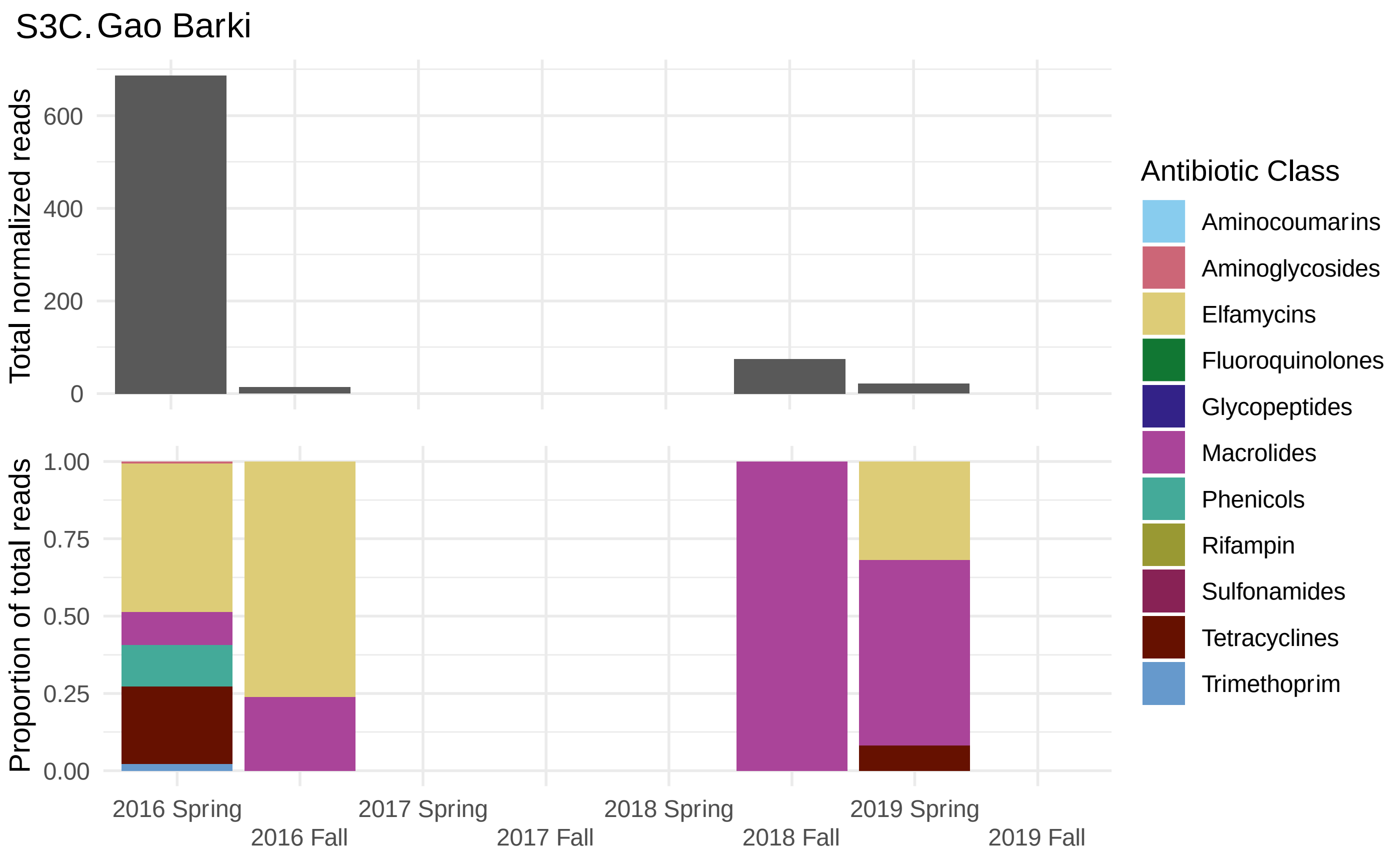
S3C. Gao Barki
Total normalized reads
600
400
200
0
Proportion of total reads
1.00
0.75
0.50
0.25
0.00
2016 Spring
2016 Fall
2017 Spring
2017 Fall
2018 Spring
2018 Fall
2019 Spring
2019 Fall
Antibiotic Class
Aminocoumarins
Aminoglycosides
Elfamycins
Fluoroquinolones
Glycopeptides
Macrolides
Phenicols
Rifampin
Sulfonamides
Tetracyclines
Trimethoprim

S3D. Soura Bildi

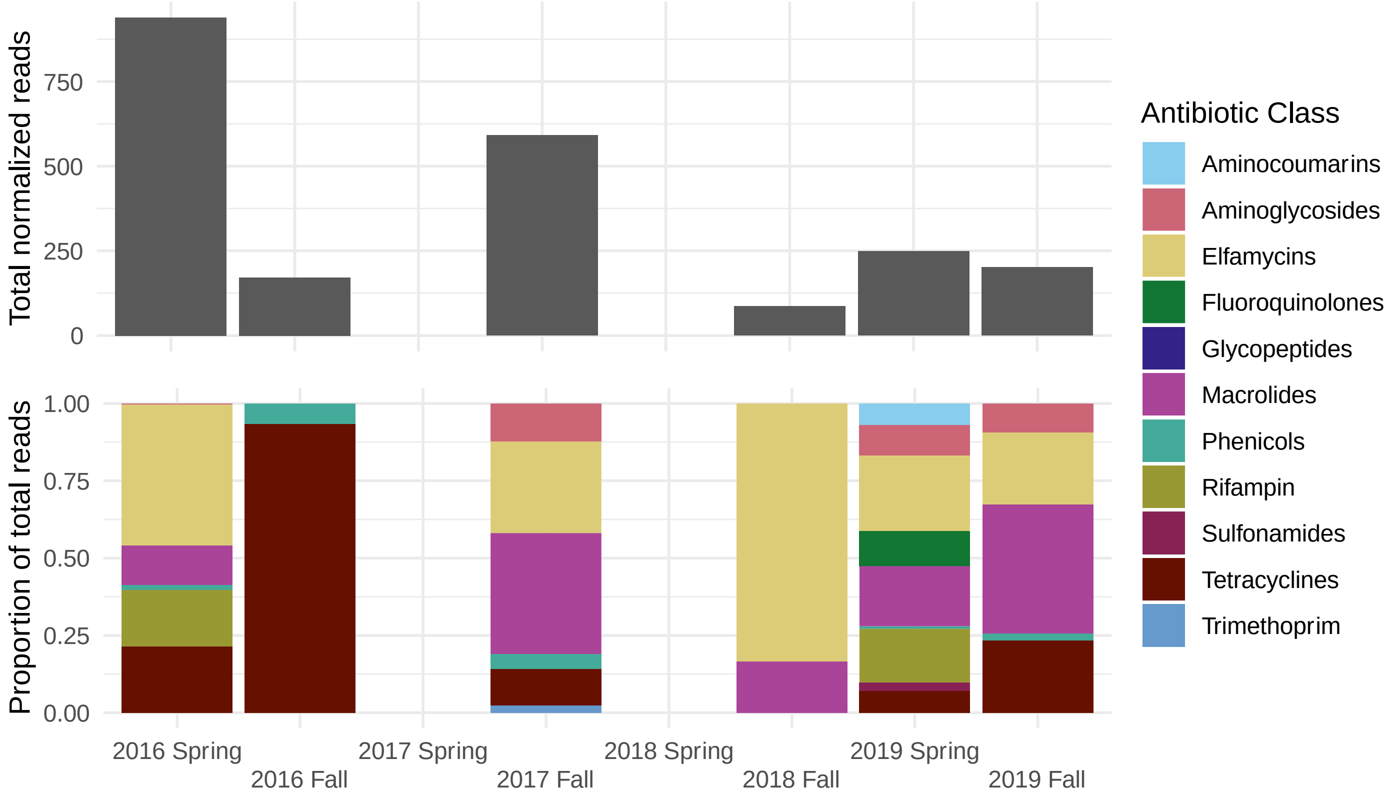

S3E. Musee National du Niger

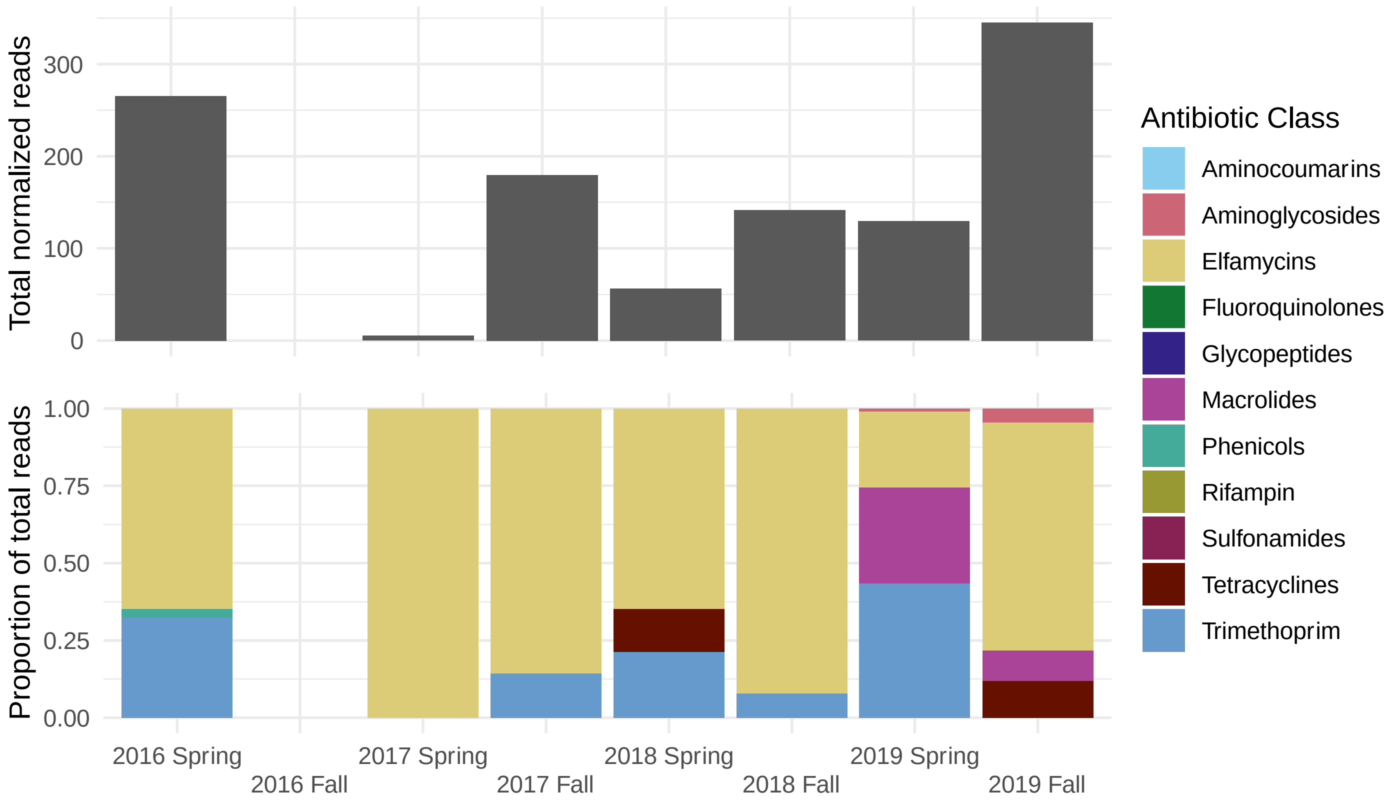

S3F. Stade Seyni Kountche

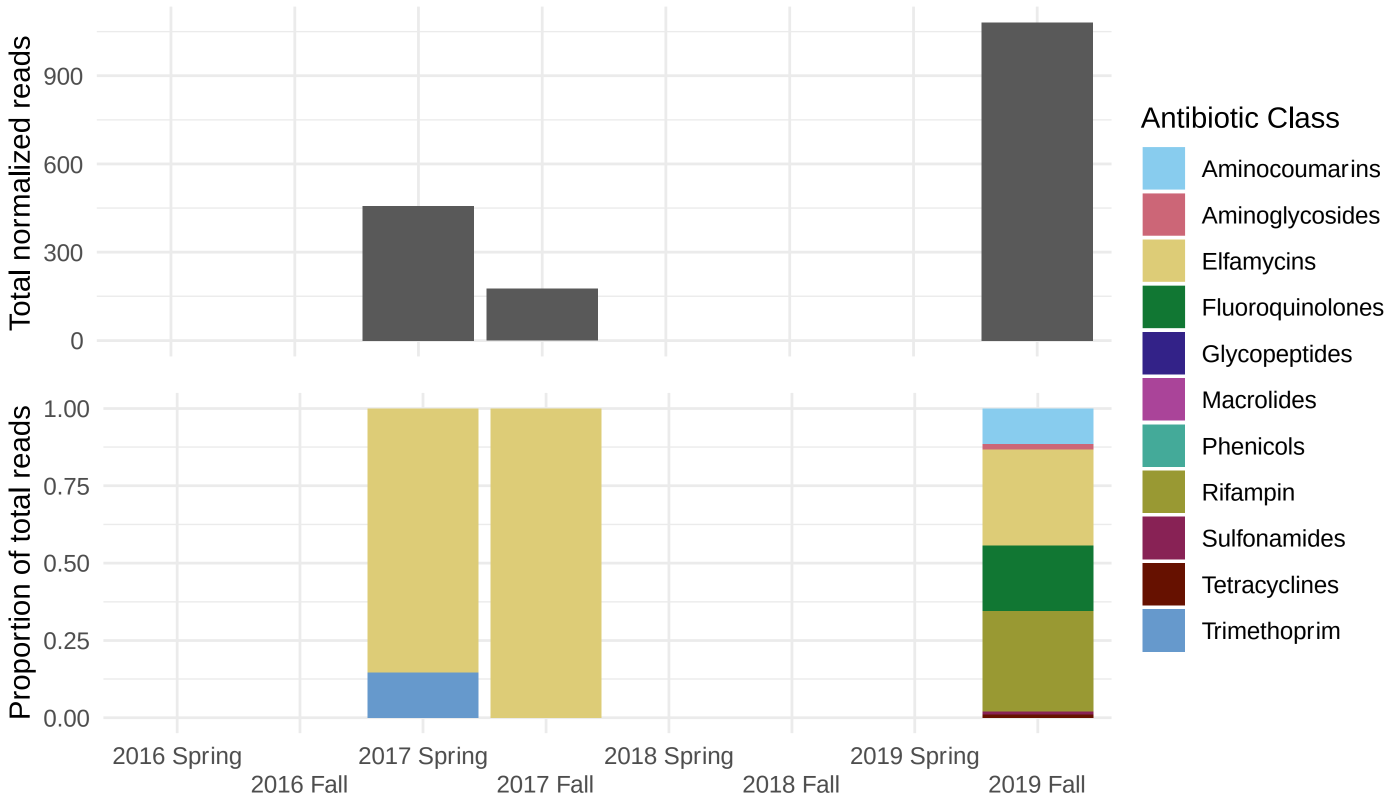

Supplement: Supplementary file 1 [file tpmd230204.SD1.pdf]
